# The Contributions of Indigenous Personality and Parenting Style to Life Satisfaction Development in Chinese Adolescents

**DOI:** 10.3389/fpsyg.2021.702408

**Published:** 2021-08-19

**Authors:** Mengting Li

**Affiliations:** Faculty of Education, The University of Hong Kong, Pok Fu Lam, Hong Kong

**Keywords:** life satisfaction, indigenous personality, adolescence, parenting styles, longitudinal

## Abstract

The present three-wave longitudinal study examined the contributions of indigenous personality traits and parenting style to life satisfaction in Chinese adolescents. Seven hundred and ten junior high school students (*M*_age_ = 11.39, *SD*_age_ = 0.53; 53.2% girls) were administered self-report measures of personality, parenting styles, and life satisfaction in Grade 6. Data on life satisfaction were collected again in Grades 7 and 8, respectively. Latent growth model analyses indicated that life satisfaction decreased over time. Regression analyses showed that the initial level of global life satisfaction was positively predicted by personality factors of dependability and interpersonal relatedness; the slope of global life satisfaction was positively predicted by personality factors of emotional stability whereas negatively predicted by interpersonal relatedness. The initial levels and slopes of different domains of life satisfaction were predicted by personality factors and parenting styles to different extents. Meanwhile, paternal and maternal parenting styles had different effects on adolescents’ life satisfaction. The study provided valuable information about the contributions of indigenous personality and both paternal and maternal parenting styles to psychological adjustment in the Chinese context. The implications of the findings concerning the associations among personality, parenting styles, and life satisfaction were discussed.

## Introduction

Life satisfaction reflects individuals’ subjective evaluations about their general satisfaction with the whole life (Diener et al., 1999). It is a crucial life outcome and has attracted many researchers’ attention over the past several decades (e.g., Casas and González-Carrasco, 2019). Previous research has shown that life satisfaction is linked to various adaptive and maladaptive functioning among adolescents (e.g., Proctor et al., 2009; Huebner et al., 2012). Given that adolescents are experiencing mental development and enduring heavy studying pressures (Arnett, 1999; Shek and Liang, 2018; Cosma et al., 2020) and that they are vulnerable to problems in affect regulation (Steinberg, 2005), it is important to deepen our knowledge of life satisfaction during the volatile adolescence period.

Studies regarding the predictors of life satisfaction are generally following two approaches: Top-down models, which assume that life satisfaction is influenced mainly by individual characteristics; and bottom-up models, which argue that environmental factors may affect life satisfaction judgments (Diener, 1984; Fan, 2014). Personality is considered a major personal determinant of individuals’ life satisfaction (e.g., Garcia, 2011). The majority of studies that adopted the top-down approach to explore the contributions of personality were based on the Big Five Model (McCrae and Costa, 2008) among adult samples (e.g., Joshanloo and Afshari, 2011). However, less research that derived from adolescents (e.g., Suldo et al., 2015; Kim et al., 2019) is not enough to reveal the relationships between personality and life satisfaction. Meanwhile, most of the previous studies regarding links between personality and life satisfaction are cross-sectional design (e.g., Xie et al., 2016), little is known about how personality could contribute to the developmental trajectories in adolescent life satisfaction.

As for the environmental factors, familial variables such as parenting styles have often been cross-sectionally examined for understanding adolescents’ life satisfaction (e.g., Milevsky et al., 2007; Di Maggio and Zappulla, 2014; Gherasim et al., 2017). Particularly, under the framework of parenting style by Baumrind (1971), authoritative parenting was associated with higher levels of life satisfaction in adolescents (e.g., Abubakar et al., 2015; Lavrič and Naterer, 2020), whereas authoritarian and permissive parenting were not reported consistent relationships with life satisfaction (e.g., Milevsky et al., 2007; Raboteg-Saric and Sakic, 2014; Abubakar et al., 2015; Xie et al., 2016; Lavrič and Naterer, 2020). Furthermore, very few studies have differentiated the functions of maternal and paternal parenting styles on life satisfaction (e.g., Milevsky et al., 2007).

In addition, culture is a pivotal factor for understanding life satisfaction in adolescents. Cultural context shapes the development trajectories of life satisfaction (e.g., Cosma et al., 2020), as well as the relationships between life satisfaction and other factors, such as personality (e.g., Kim et al., 2018) and parenting styles (e.g., Lavrič and Naterer, 2020). For example, while adolescents in some cultural settings displayed decreased trends in life satisfaction, stable or increased patterns in life satisfaction were viewed in adolescents in some other cultural settings (e.g., Cosma et al., 2020). Furthermore, the relationships between the Big Five personality and adolescents’ life satisfaction were shown to be different across cultures (e.g., Kim et al., 2018), and the relationships of authoritarian and permissive parenting to life satisfaction varied across cultures (e.g., Lavrič and Naterer, 2020). Particularly, despite such cultural variation, the majority of studies investigated the change trend in life satisfaction or examined the predictors of life satisfaction in Western settings (e.g., Martin et al., 2008; Suldo et al., 2015), not in non-western backgrounds such as China.

Some culture-specific contents in personality that are emphasized in the Chinese cultural context are not reflected in the Big Five Model, such as interpersonal relatedness which reflects the Chinese indigenous values of family orientation and harmony (Yang, 2006; Cheung et al., 2008; Fan et al., 2011). Meanwhile, unlike the Western cultures that emphasize independence and autonomy, in China, authoritarian parenting with strictness and authority is considered as a signal of parental involvement and deemed beneficial for children (Chao, 1994). Accordingly, cultural variation in the relationships of personality and parenting to life satisfaction may exist between Chinese and Western backgrounds. However, the contributions of personality and parenting styles to adolescent life satisfaction development are yet to be understood in Chinese context.

To fill out these research gaps and examine the differential contributions of personal and familial factors to adolescent life satisfaction, the present three-wave longitudinal study investigated the predictors of changes in life satisfaction in Chinese adolescents. In this study, the influences of both maternal and paternal parenting styles and both the universal personality and Chinese indigenous personality on adolescents’ life satisfaction developmental trajectories were explored as well. In addition, because global life satisfaction and domain-specific satisfaction are distinguishable from each other (e.g., Weber and Huebner, 2015), the contributions of personality and parenting styles to global and specific domain-specific satisfaction were considered separately in this study.

### The Development of Life Satisfaction in Adolescents

Even though there are longitudinal studies examining the change of adolescents’ life satisfaction, inconsistent findings have generally been showed in the literature. For example, Shoshani and Slone (2013) found that Israeli adolescents showed lower levels of life satisfaction in their eighth grade than in the seventh grade. A similar decrease pattern in life satisfaction was also reported in Spanish adolescents (Gomez-Baya et al., 2018), Korean adolescents (Jung and Choi, 2017), and American adolescents (Martin et al., 2008). In contrast, Lewis et al. (2011) found that US middle school students’ life satisfaction significantly increased over 5 months. In a 2-year longitudinal study, Salmela-Aro and Tuominen-Soini (2010) reported that life satisfaction increased during the transition from Grade 9 to upper secondary or vocational education among Finnish adolescents. In addition, some other studies showed a stable pattern of life satisfaction during adolescence. For example, Marques et al. (2011, 2013) reported non-significant changes in life satisfaction in Portuguese adolescents over 1- and 2-year intervals, respectively. Bratt (2015) found that life satisfaction was stable over 3 years among Norwegian middle school students.

Under cross-cultural backgrounds, the results of change in adolescents’ life satisfaction have also been reported (e.g., Cosma et al., 2020; Marquez and Long, 2020). For example, based on the data from the International Health Behavior survey between 2002 and 2018 among adolescents in 36 countries, Cosma et al. (2020) found adolescents from 13 countries had a decreasing trend in life satisfaction (e.g., Greece, Austria, and Canada), and adolescents from the other 13 countries had increases in life satisfaction (e.g., Romania, Croatia, and Lithuania), whereas the adolescents from the rest 10 countries were stable in life satisfaction (e.g., Germany, France, and Italy). Based on the data from the Program for International Student Assessment, Marquez and Long (2020) analyzed the change trends of life satisfaction in 15-year-old adolescents between 2015 and 2018 in 46 countries. In their article, a decline trend of life satisfaction among adolescents in 39 out of 46 countries (e.g., France, Germany, and Russia) was reported. Adolescents in seven countries (e.g., Thailand, Spain, and Italy) had a stable level of life satisfaction between 2015 and 2018. Only South Korean adolescents showed an increase in life satisfaction during the period.

As for studies in Chinese context, most of them have been conducted among Hong Kong and Taiwanese adolescents. For example, Leung et al. (2004) found that the global life satisfaction of Hong Kong adolescents significantly declined from the beginning to the end of the 7th grade. Also in Hong Kong adolescents, Shek and Liang (2018) reported that life satisfaction showed a declining trend in a 6-year period. Jhang (2018) found that life satisfaction declined across 2 years among Taiwanese junior high school students in poverty. In addition, two inconsistent findings were reported for the adolescents from mainland China. One was conducted by Wang and Zhang (2012), and a decrease pattern in life satisfaction from Grade 7 to Grade 9 was reported. The other was performed by Nie et al. (2019) and they found life satisfaction displayed a stable pattern across 2 years among high school students.

To sum up, all of those above-mentioned inconsistent research findings suggest more longitudinal studies need to be conducted to explore the development trajectories in life satisfaction among adolescents.

### Life Satisfaction and Personality

Compared with studies exploring links between personality and life satisfaction in adults, there are fewer studies conducted in adolescent samples (Anglim et al., 2020). Furthermore, the majority of studies that examined the association between personality and life satisfaction in adolescents were in light of the Big Five Model. For instance, neuroticism has consistently been shown to be the strongest predictor of life satisfaction and was negatively related to life satisfaction (e.g., Suldo et al., 2015; Xu et al., 2017). Extraversion and conscientiousness were found to be positively associated with life satisfaction (e.g., Suldo et al., 2015; Weber and Huebner, 2015). As for openness, most empirical studies reported a non-significant relationship between openness and life satisfaction (e.g., Jovanović, 2019; Kim et al., 2019). Only a few found a positive link between openness and life satisfaction (e.g., Suldo et al., 2015). The relationship between agreeableness and life satisfaction was also inconsistent in the literature. Some showed a positive link between agreeableness and life satisfaction (e.g., Jovanović, 2019), whereas others reported non-significant relationships (e.g., Marcionetti and Rossier, 2016).

Despite such research findings about personality and life satisfaction in adolescents, with very few exceptions (e.g., Weber and Huebner, 2015), previous studies have mostly focused on the relationships between personality and global life satisfaction, so that little is known about the relationships between personality and specific domains of life satisfaction. For example, among US adolescents, Weber and Huebner (2015) found that functions of personality were varied in different domains of life satisfaction. Specifically, neuroticism negatively predicted all five domains of satisfaction (i.e., family life, friendships, school experiences, self, and living environment). Extraversion was positively related to satisfaction with friendships, self, and living environment. Openness was positively associated with satisfaction with family life, school experiences, and self. Agreeableness was only positively linked to satisfaction with family life. Conscientiousness was positively related to all life satisfaction domains except for satisfaction with friendships.

As shown in the above literature review, the relationship between personality (mainly in terms of the Big Five dimensions) and life satisfaction including global and specific domains has been explored mainly in western backgrounds. However, because the Big Five Model is derived from the western contexts, it may not be sufficient to capture some non-western personality features in other cultural contexts (Yang, 2006; Cheung et al., 2008; Fan et al., 2011). Accordingly, the association between personality and life satisfaction in non-western backgrounds may show very distinguished patterns from those in western backgrounds. Furthermore, the levels of global life satisfaction and the levels of satisfaction in various domains are not necessarily consistent (e.g., Jovanović et al., 2017), and the patterns of associations between global or different domain-specific satisfaction and personality are different (e.g., Lent et al., 2005; Weber and Huebner, 2015). Therefore, more empirical studies in non-western settings may further increase our knowledge about the relations of personality to both global and domain-specific life satisfaction across cultures.

For example, in the Chinese context in which Confucian philosophy is highlighted, there are a few culture-specific personality contents that have not been covered in the Big Five Model, such as harmony, face, Renqing, and family orientation (Cheung et al., 2008). Those Chinese indigenous personality attributes in adolescents have been systematically and effectively assessed by the factor of interpersonal relatedness of the Cross-Cultural (Chinese) Personality Assessment Inventory for Adolescents (CPAI-A), which is derived from Chinese settings and has been validated across eastern and western cultural settings (Fan et al., 2011; Cheung et al., 2013). In addition, the CPAI-A also includes universal independent personality factors such as social potency, emotional stability, and dependability that are correspondingly correlated with those factors in the Big Five model (Cheung et al., 2001, 2003; Lin and Church, 2004).

Empirical studies have examined the association between the personality dimensions of CPAI and life satisfaction in the Chinese samples (e.g., Chen et al., 2006; Zhang et al., 2011). For example, in a sample of Hong Kong adolescents, Ho et al. (2008) reported that certain indicators of social potency, emotional stability, and interpersonal relatedness positively predicted life satisfaction. Among junior secondary school students, Xie et al. (2016) reported positive relationships of emotional stability, dependability, and interpersonal relatedness to global life satisfaction. In addition, they also found that different domains of life satisfaction were predicted by different personality dimensions. However, all those aforementioned studies are cross-sectional design and did not provide evidence for the contributing roles of CPAI dimensions on the development trajectories of adolescents’ life satisfaction.

### Life Satisfaction and Parenting Styles

According to Baumrind (1991), three distinct styles could be identified based on the demandingness (also referred to as behavioral control) and responsiveness (also referred to as warmth) of parenting: authoritative, authoritarian, and permissive parenting. Authoritative parenting emphasizes clear standards and support and is characterized by being both demanding and responsive based on children’s needs and capabilities. Authoritarian parenting emphasizes order and obedience and is characterized by demanding parental behaviors but without responsiveness. Permissive parenting has more responsiveness than demandingness, and parents with this style allow children’s self-regulation and do not accentuate authority. This model of parenting styles has been widely examined across cultures in the past 40 years (e.g., Xie et al., 2016).

For many years, studies regarding the relationships of parenting styles to life satisfaction have mostly been conducted in children (Di Maggio and Zappulla, 2014). Recently, researchers have begun to notice the impact of parenting practices on life satisfaction in adolescence (e.g., Coccia et al., 2012). Empirical studies provided evidence on the links between parenting styles and adolescents’ life satisfaction across Western (e.g., Milevsky et al., 2007) and non-Western contexts (e.g., Abubakar et al., 2015).

Higher authoritative parenting was often found to contribute to higher levels of life satisfaction in both Western and non-Western cultures (e.g., Milevsky et al., 2007; Xie et al., 2016). However, the influence of authoritarian parenting was shown to be different across cultures. In the United States (e.g., Milevsky et al., 2007), Romanian (e.g., Gherasim et al., 2017), and Russian contexts (e.g., Gherasim et al., 2017), authoritarian parenting was related to lower levels of life satisfaction. However, Xie et al. (2016) reported a positive link between authoritarian parenting style and school satisfaction in a group of Chinese adolescents. In other contexts, such as Indonesian (e.g., Abubakar et al., 2015) and French (e.g., Gherasim et al., 2017) contexts, no significant relationship between authoritarian and life satisfaction was found.

As for permissive parenting style, Xie et al. (2016) and Lavrič and Naterer (2020) reported that permissive parenting was positively associated with life satisfaction in Chinese adolescents and Romanian youths, respectively. Raboteg-Saric and Sakic (2014) found that Croatian adolescents with permissive mothers and fathers are more satisfied with their life than those with authoritarian mothers and fathers. However, negative associations between permissive parenting and life satisfaction among Albanian, Bosnian, Croatian, and Kosovo youths were reported in the literature (Lavrič and Naterer, 2020).

In addition, it has been shown that the functions of maternal parenting style and paternal parenting style on offspring’s life satisfaction are different (e.g., Milevsky et al., 2007). For example, Abubakar et al. (2015) reported that Indonesian adolescents’ life satisfaction was only positively predicted by paternal authoritativeness, but not by maternal authoritativeness. Milevsky et al. (2007) found that US adolescents with maternal authoritative style had higher life satisfaction than those with maternal permissive styles, whereas no significant difference was found in life satisfaction between adolescents with paternal authoritative style and those with paternal permissive styles.

The influence of parenting styles on Chinese adolescents’ life satisfaction was supported in the literature. For example, in addition to the study by Leung et al. (2004) and Xie et al. (2016) reported that maternal restrictiveness (also known as demandingness) was negatively associated with Hong Kong adolescents’ family, self, and friend satisfaction 8 months later; and maternal concern (also known as responsiveness) was positively correlated with family, self, and friend satisfaction 8 months later. Xiang et al. (2017) reported that parental autonomy support positively predicted adolescents’ life satisfaction, and psychological control negatively predicted adolescents’ life satisfaction. In a longitudinal study with 1 year interval, Shek (2007) found that paternal and maternal psychological control negatively predicted Hong Kong adolescents’ life satisfaction. Gao et al. (2021) reported that paternal and maternal warmth positively related to adolescents’ life satisfaction 8 months later. Zhu and Shek (2021) found that trajectories of paternal and maternal behavioral control positively predicted trajectory of adolescents’ life satisfaction, and paternal and maternal psychological control negatively predicted trajectory of adolescents’ life satisfaction during the 6-year high school period. However, with the exception of Zhu and Shek’s (2021) work, most of these studies are cross-sectional or short-term longitudinal design, and the antecedents to the development trajectories of life satisfaction cannot be well examined in Chinese adolescents up to now. Meanwhile, none of them has explored functions of maternal and paternal parenting styles on different aspects of life satisfaction separately.

### The Present Study

In response to the abovementioned research gaps for the contributions of personality and parenting styles in Chinese adolescents, the present three-wave longitudinal study aimed at the following three points. The first was to investigate the stability and change in life satisfaction in adolescents from mainland China. Based on the previous research findings (e.g., Wang and Zhang, 2012; Shek and Liang, 2018), we expected decreasing trends in life satisfaction during the present study period. Second, the influence of both universal and indigenous personalities on adolescents’ development trajectories in life satisfaction was explored. Given the findings regarding the significant links between CPAI dimensions and life satisfaction (e.g., Xie et al., 2016), we expected that higher levels of social potency, dependability, emotional stability, and interpersonal relatedness would relate to higher levels of adolescents’ life satisfaction.

Third, in terms of Baumrind’s (1991) framework of parenting styles, the contributions of parenting styles to the development trajectories of life satisfaction during adolescence were investigated as well. Consistent with previous relevant findings (e.g., Xie et al., 2016; Gherasim et al., 2017), I hypothesized that perceived authoritative and permissive parenting would positively relate to life satisfaction. Due to the inconsistent research findings in the literature (e.g., Abubakar et al., 2015; Xie et al., 2016), no specific hypothesis was made regarding the influence of authoritarian parenting on life satisfaction. In addition, given previous supporting on the different effects of paternal parenting and maternal parenting on adolescents’ life satisfaction, (e.g., Abubakar et al., 2015), I further hypothesized that paternal and maternal parenting styles would have different functions on Chinese adolescents’ life satisfaction.

## Materials and Methods

### Participants

Participants in this study were students from four public junior high schools in Shanghai, China. The initial sample consisted of 710 students (*M*_age_ = 11.39, *SD*_age_ = 0.53; 53.2% girls) in Grade 6. Six hundred sixty-one students in Grade 7 (93.10%) participated in the second wave of data collection, and 646 (90.10%) students in Grade 8 participated in the third wave of data collection. All participants received their personality profiles at the end of the longitudinal study as reward. Results from attrition analyses indicated non-significant differences among all study variables (i.e., parenting styles, personality, and life satisfaction) at Grade 6 between students who participated in all three waves and those who participated in only one or two waves: Wilks’ Λ = 0.97, *F* (16, 596) = 1.01, *p* = 0.45. Consent forms were obtained from both participants and their parents. The majority (90.6% fathers and 87.4% mothers) of the participants’ parents had a high school or higher degree.

### Measurements

#### Personality

The Cross-Cultural (Chinese) Personality Inventory for Adolescents (CPAI-A; Form B; Cheung et al., 2008) was used to measure both universal and indigenous personality of participants. It is a self-report measure that features a yes-or-no format with a total of 307 items. The CPAI-A (Form B) consists of four factors encompassing 25 personality scales: Social Potency, Dependability, Emotional Stability, and Interpersonal Relatedness. Social Potency is related to Extraversion and Openness in the Big Five model and refers to orientation toward novelty, change, self-development, and sociability (e.g., “I do not like stable jobs; instead, I like challenges.”). Dependability is similar to Conscientiousness and comprises responsibility, discipline, and meaning in life (e.g., “I make good use of my time after school to learn different things so as to enrich my life”). Emotional Stability is related to Neuroticism and consists of emotionality, inferiority versus self-acceptance, optimism versus pessimism, and face (e.g., “Sometimes I feel miserable for no reason”). The Interpersonal Relatedness factor consists of the most typical interdependent dispositions in collectivistic cultures such as Renqing (Relationship Orientation), Harmony, and Family Orientation (e.g., “I do not mind suffering a bit of loss as long as it can prevent disputes”). Factor level of personality measured was employed in the current study. Each factor score is the average score of the corresponding subscales. A number of studies have reported good internal consistency reliability, test-retest reliability, and construct validity of the CPAI-A (Form B; Cheung et al., 2008; Li et al., 2019). The structural validity of CPAI-A in the present study was tested by CFA (because Social Potency and Interpersonal Relatedness factors includes more than five subscales, subscales under these two factors were parceled into three parcels using random assignment) and results indicated adequate model fit indices: χ^2^ = 343.80, *df* = 72, CFI = 0.90, RMSEA = 0.073, SRMR = 0.072.

#### Parenting Styles

The Parental Authority Questionnaire (Buri, 1991) was used to measure paternal and maternal authoritarian (10 items; e.g., “As I was growing up my father (mother) often told me exactly what he (she) wanted me to do and how he (she) expected me to do it”), authoritative (10 items; e.g., “As I was growing up my father (mother) gave me clear direction for my behaviors and activities but he(she) was also understanding when I disagreed with him (her)”), and permissive (10 items; e.g., “My father (mother) did not view himself (herself) as responsible for directing and guiding my behavior as I was growing up”) parenting styles. Adolescents were asked to rate the total 60 items on a 5-point scale, ranging from 1 (not at all true) to 5 (very true) for their mother (30 items) and father (30 items). The PAQ has good psychometric properties in the Chinese context (e.g., Xie et al., 2016). The structural validity of PAQ in the present study was tested by CFA (items under each factor were parceled into three parcels using random assignment) and results indicated adequate model fit indices for maternal styles (χ^2^ = 114.87, *df* = 22, CFI = 0.95, RMSEA = 0.079, SRMR = 0.050) and paternal styles (χ^2^ = 105.48, *df* = 22, CFI = 0.96, RMSEA = 0.075, SRMR = 0.049).

#### Life Satisfaction

Life satisfaction was measured by the Chinese Adolescents’ Life Satisfaction Scale (CALSS; Cheung and Cheung, 2005), which was developed based on the Satisfaction with Life Scale (Diener et al., 1985) and the Multidimensional Students’ Life Satisfaction Scale (Gilman et al., 2000). The 30-item CALSS assessed both the global life satisfaction (5 items; e.g., “I am satisfied with my life”), and the satisfaction in five specific domains: family (7 items; e.g., “My parents could understand me very well”), friend (4 items; e.g., “I have a number of good friends”), health (4 items; e.g., “My body is very healthy”), school (9 items; e.g., “Most classmates do not like me”), and self (6 items; e.g., “I am confident very much”). Adolescents were asked to rate each item on a 7-point scale, ranging from 1 (not at all true) to 7 (very true). Previous studies reported good psychometric properties of this inventory in the Chinese context (e.g., Ho et al., 2010).

### Data Analysis

A power analysis was conducted to determine whether the sample size of this study is enough to do the following analysis using G^∗^power. According to previous study regarding the influences of personality and parenting styles on life satisfaction (e.g., Xie et al., 2016), the *R*^2^ was set to 0.30, the power was set to 0.80 (Faul et al., 2009), alpha was set to 0.05, the number of predictors was set to 10. Results of the power analysis suggested to collect data on a sample of 389 participants, which is lower than the actual number of participants (*N* = 710) in this study.

The scalar invariance is a prerequisite for comparing means across different time points (Klimstra et al., 2018). In order to employ latent growth models, the scalar invariance of life satisfaction was examined. Because item parceled solution, relative to the individual item solution, resulted in less bias in estimates of structural parameters (Bandalos, 2002), items under the dimensions with more than five items (i.e., family satisfaction, school satisfaction, and self-satisfaction) were parceled into three parcels using random assignment (Little et al., 2002). Overall, evidence for scalar invariance in global and four domain-specific life satisfactions (except for self-satisfaction) was found, which implies that valid conclusions from growth curve models based on observed variables could be drawn. Therefore, latent growth models based on observed variables were employed to explore the change trajectories of global, family, friend, health, and school satisfaction.

As for the self-satisfaction, although full scalar invariance was not supported, evidence for the partial scalar invariance (i.e., with at least one of the intercepts constrained to be equal across time) can still support the comparison of latent means across time (Steinmetz, 2013). Therefore, latent growth models based on latent variables with partial scalar invariance constraints that were imposed on the confirmatory factor analysis models for measurement invariance were employed to explore the change trajectories of self-satisfaction.

The skewness (ranged from −1.37 to 0.33) and kurtosis (−0.69 to 1.25) values of the research variables indicated the normal distributions of the data (Kline, 1998). Zero-order correlational analysis was conducted. Repeated measure multivariate analysis of variance (MANOVA) was used to examine the time and gender differences of life satisfaction. Another MANOVA was used to examine the gender difference in personality and parenting styles. Latent growth models were employed to examine the change trajectories of life satisfaction with the maximum likelihood estimator. Missing data were handled in M*plus* using full information maximum likelihood estimation. Because intercept and linear slope were examined with a three-wave data set in the current study, the degree of freedom (*df*) should be 1. However, as argued by researchers (Kenny et al., 2014; Taasoobshirazi and Wang, 2016), it may be problematic and possibly misleading to use Root Mean Square Error of Approximation (RMSEA) as a fit index when *df* equals 1. As such, only Comparative Fit Index (CFI) and Standardized Root Mean Square Residual (SRMR) were fit indices referred to the latent growth model analysis. Regression paths were then added into the growth models to examine the influence of personality and parenting styles on the growth patterns of life satisfaction using M*plus*.

In terms of the common method bias (CMB), Harman’s single factor was used to test the potential limitation of the self-reported personality, parenting styles, and life satisfaction. The total variance for a single factor is 10.60%, suggesting an acceptable rate that is lower than 50% (Podsakoff et al., 2003).

## Results

### Preliminary Analysis

Descriptive statistics of the study variables, their correlations, and Cronbach’s alphas are presented in Tables 1 and 2. The overall effects of time, gender, and their interactions on life satisfaction in three waves were examined through mixed repeated MANOVA. According to Cohen’s (1988) guidelines for interpreting *F*-test effect size (small = 0.01, medium = 0.059, and large = 0.138), only time difference, Wilks’ Λ = 0.85, *F* (12, 589) = 8.70, *p* = 0.00, η^2^ = 0.15 was further considered. The overall effect of gender on personality and parenting styles was examined through another MANOVA. According to the Cohen’s (1988) guidelines, gender difference was not considered in the following analyses. Correlation results showed that, in general, social potency, dependability, emotional stability, interpersonal relatedness, and authoritative parenting style were positively correlated with life satisfaction. Authoritarian parenting style was negatively correlated with life satisfaction. Permissive parenting style was positively correlated with family, self, and global satisfaction.

**TABLE 1:** Descriptive statistics and zero-order correlations among personality, parenting styles, and life satisfaction at Time 1.

		***M***	***SD***	**1**	**2**	**3**	**4**	**5**	**6**	**7**	**8**	**9**	**10**	**11**	**12**	**13**	**14**	**15**	**16**
1	CPAI_SP	7.36	1.49	(0.84)															
2	CPAI_DEP	7.51	2.01	0.45**	(0.79)														
3	CPAI_ES	8.40	2.26	0.43**	0.49**	(0.86)													
4	CPAI_IR	8.40	1.78	0.38**	0.40**	0.73**	(0.88)												
5	AN_F	2.80	0.81	−0.15**	−0.14**	−0.21**	−0.23**	(0.82)											
6	AU_F	3.72	0.75	0.27**	0.31**	0.27**	0.28**	−0.24**	(0.83)										
7	Per_F	3.08	0.59	0.09**	0.10*	0.08*	0.02	0.03	0.50**	(0.63)									
8	AN_M	2.85	0.82	−0.11**	−0.11**	−0.23**	−0.25**	0.82**	−0.20**	0.07	(0.83)								
9	AU_M	3.77	0.73	0.23**	0.27**	0.24**	0.29**	−0.22**	0.80**	0.39**	−0.26**	(0.81)							
10	Per_M	3.08	0.60	0.05	0.07	0.05	–0.01	0.07	0.33**	0.81**	0.05	0.43**	(0.63)						
11	Family_T1	5.57	1.07	0.28**	0.36**	0.40**	0.43**	−0.46**	0.53**	0.24**	−0.46**	0.54**	0.22**	(0.78)					
12	Friend_T1	6.04	1.13	0.37**	0.26**	0.30**	0.34**	−0.28**	0.30**	0.04	−0.23**	0.28**	0.01	0.45**	(0.76)				
13	Health_T1	5.19	1.47	0.23**	0.15**	0.22**	0.16**	−0.12**	0.12**	0.07	−0.10**	0.11**	0.03	0.25**	0.21**	(0.75)			
14	School_T1	5.61	1.01	0.33**	0.41**	0.41**	0.41**	−0.26**	0.35**	0.03	−0.26**	0.34**	–0.02	0.49**	0.54**	0.25**	(0.79)		
15	Self_T1	4.95	1.25	0.39**	0.29**	0.29**	0.21**	–0.04	0.34**	0.18**	–0.04	0.32**	0.16**	0.40**	0.48**	0.24**	0.31**	(0.80)	
16	Global_T1	4.81	1.23	0.28**	0.31**	0.31**	0.36**	−0.10**	0.38**	0.24**	−0.09*	0.36**	0.22**	0.50**	0.36**	0.21**	0.35**	0.47**	(0.67)

CPAI_SP, Social Potency; CPAI_DEP, Dependability; CPAI_ES, Emotional Stability; CPAI_IR, Interpersonal Relatedness; An_F, Paternal Authoritarian Style. Au_F, Paternal Authoritative Style; Per_F, Paternal Permissive Style; An_M, Maternal Authoritarian Style; Au_M, Maternal Authoritative Style; Per_M, Maternal Permissive Style. Family, Friend, Health, School, Self, Global means domain-specific life satisfaction and global satisfaction. The Cronbach’s alphas are shown on the diagonal line.

*p < 0.05; **p < 0.01.

**TABLE 2:** Descriptive statistics of life satisfaction at Time 2 and Time 3 and their zero-order correlations with personality and parenting styles.

	***M***	***SD***	**α**	**CPAI_SP**	**CPAI_DEP**	**CPAI_ES**	**CPAI_IR**	**AN_F**	**AU_F**	**Per_F**	**AN_M**	**AU_M**	**Per_M**
Family_T2	5.26	1.16	0.82	0.21**	0.26**	0.35**	0.36**	−0.28**	0.40**	0.19**	−0.33**	0.43**	0.15**
Friend_T2	5.78	1.26	0.81	0.32**	0.26**	0.32**	0.35**	−0.14**	0.30**	0.08*	−0.17**	0.29**	0.06
Health_T2	5.04	1.43	0.74	0.18**	0.15**	0.21**	0.18**	−0.11**	0.14**	0.02	−0.12**	0.14**	–0.01
School_T2	5.32	1.03	0.79	0.29**	0.40**	0.40**	0.41**	−0.10*	0.31**	0.03	−0.13**	0.29**	–0.01
Self_T2	4.78	1.38	0.86	0.36**	0.27**	0.36**	0.25**	–0.05	0.30**	0.12**	−0.10**	0.29**	0.10**
Global_T2	4.62	1.35	0.75	0.21**	0.27**	0.34**	0.32**	−0.13**	0.29**	0.14**	−0.14**	0.31**	0.15**
Family_T3	5.25	1.13	0.86	0.21**	0.26**	0.33**	0.33**	−0.31**	0.41**	0.15**	−0.32**	0.42**	0.14**
Friend_T3	5.74	1.12	0.80	0.26**	0.25**	0.31**	0.30**	−0.19**	0.31**	0.09*	−0.19**	0.32**	0.07
Health_T3	4.98	1.33	0.76	0.19**	0.14**	0.19**	0.18**	–0.05	0.15**	0.00	–0.08	0.18**	0.01
School _T3	5.29	0.96	0.81	0.23**	0.31**	0.32**	0.33**	−0.15**	0.29**	0.01	−0.16**	0.30**	0.01
Self_T3	4.77	1.34	0.88	0.31**	0.28**	0.32**	0.24**	−0.13**	0.25**	0.04	−0.14**	0.24**	0.03
Global_T3	4.53	1.24	0.77	0.21**	0.33**	0.37**	0.31**	−0.17**	0.24**	0.03	−0.14**	0.25**	0.04

CPAI_SP, Social Potency; CPAI_DEP, Dependability; CPAI_ES, Emotional Stability; CPAI_IR, Interpersonal Relatedness; An_F, Paternal Authoritarian Style; Au_F, Paternal Authoritative Style; Per_F, Paternal Permissive Style; An_M, Maternal Authoritarian Style; Au_M, Maternal Authoritative Style; Per_M, Maternal Permissive Style. Family, Friend, Health, School, Self, Global means domain-specific life satisfaction and global satisfaction.

*p < 0.05; **p < 0.01.

### Growth Model Analyses of Life Satisfaction

Latent growth models were conducted to examine the linear changes in life satisfaction. The model fit indices (i.e., CFI and SRMR) indicated adequate data fit for all linear growth models except for self-satisfaction (Table 3). The latent change model for self-satisfaction was not convergent. Therefore, no model fit indices for self-satisfaction were reported. Results revealed that life satisfaction (except for self-satisfaction) had medium-high initial levels that significantly decreased over time. The significant negative intercept-slope correlations in friend, school, and global satisfaction indicated that adolescents who initially scored higher reported a sharper decrease with time in the four aforementioned life satisfactions.

**TABLE 3:** Latent growth models for life satisfaction.

	**Growth factors**			**Model fit indices**			
	**Intercept (I)**	**Slope (S)**	***r*(I, S)**	**χ^2^/*df***	**CFI**	**RMSEA**	**SRMR**
	*M*(σ^2^)	*M*(σ^2^)					
Family	5.54**(0.76**)	−0.17**(0.14**)	–0.08	15.368/1	0.975	0.142	0.028
Friend	6.02**(0.80**)	−0.15**(0.19**)	−0.15**	5.557/1	0.988	0.080	0.019
Health	5.17**(1.16**)	−0.10**(0.15*)	–0.14	0.331/1	1.000	0.000	0.005
School	5.58**(0.71**)	−0.16**(0.13**)	−0.12**	15.877/1	0.973	0.145	0.028
Global	4.81**(0.97**)	−0.15**(0.26**)	−0.23**	1.081/1	1.000	0.011	0.009

Family, Friend, Health, School, Global means domain-specific life satisfaction and global satisfaction.

*p < 0.05; **p < 0.01.

### Regression Analyses on Personality and Parenting Styles Predicting Life Satisfaction

Five models were then tested with the growth patterns of life satisfaction regressing on personality and parenting styles to examine the predicting roles of personality and parenting styles in global and four domain-specific life satisfactions except for self-satisfaction (see Table 4). All five models indicated good fit index with χ^2^/*df* smaller than 5, CFI larger than 0.90, and RMSEA smaller than 0.08, SRMR smaller than 0.08 (Hu and Bentler, 1999; Kline, 2005).

**TABLE 4:** Regression analyses for predicting growth of life satisfaction by personality and parenting style.

	**Family satisfaction**				**Friend satisfaction**				**Health satisfaction**					
	**Intercept**	***sr*^2^**	**Slope**	***sr*^2^**	**Intercept**	***sr*^2^**	**Slope**	***sr*^2^**	**Intercept**	***sr*^2^**	**Slope**	***sr*^2^**		
SP	−0.01(0.80) [−0.09,0.07]	0.000	−0.02(0.75) [−0.16,0.12]	0.000	0.30(0.00) [0.19,0.41]	0.038	−0.22(0.00) [−0.36, −0.07]	0.012	0.17(0.00) [0.05,0.30]	0.014	−0.09(0.37) [−0.64,0.47]	0.001		
DEP	0.14(0.00) [0.06,0.22]	0.008	−0.12(0.08) [−0.25,0.02]	0.003	0.06(0.23) [−0.04,0.16]	0.002	0.01(0.92) [−0.14,0.15]	0.000	−0.00(0.98) [−0.12,0.12]	0.000	0.01(0.92) [−0.38,0.41]	0.000		
ES	0.06(0.21) [−0.04,0.16]	0.003	0.12(0.16) [−0.06,0.29]	0.004	−0.01(0.87) [−0.17,0.14]	0.000	0.20(0.03) [0.003,0.39]	0.008	0.19(0.02) [0.03,0.36]	0.009	−0.12(0.37) [−0.59,0.36]	0.001		
IR	0.19(0.00) [0.09,0.29]	0.012	−0.10(0.22) [−0.27,0.07]	0.001	0.20(0.00) [0.07,0.34]	0.011	−0.15(0.09) [−0.31,0.01]	0.003	−0.01(0.88) [−0.17,0.15]	0.000	0.05(0.71) [−0.35,0.44]	0.000		
AN_F	−0.21(0.00) [−0.34, −0.07]	0.007	0.19(0.07) [−0.10,0.49]	0.003	−0.19(0.02) [−0.35, −0.03]	0.005	0.23(0.04) [0.005,0.51]	0.004	−0.05(0.63) [−0.25,0.16]	0.000	0.16(0.32) [−0.41,0.73]	0.002		
AU_F	0.16(0.02) [0.01,0.32]	0.006	0.08(0.45) [−0.17,0.34]	0.001	0.18(0.04) [0.01,0.43]	0.006	0.00(0.98) [−0.28,0.29]	0.000	−0.01(0.96) [−0.21,0.20]	0.000	0.04(0.82) [−0.56,0.64]	0.000		
Per_F	0.01(0.89) [−0.15,0.17]	0.000	−0.05(0.70) [−0.31,0.22]	0.000	−0.09(0.36) [−0.29,0.12]	0.001	0.15(0.22) [−0.19,0.50]	0.001	0.18(0.11) [−0.05,0.40]	0.001	−0.33(0.08) [−1.20,0.54]	0.006		
AN_M	−0.17(0.01) [−0.30, −0.03]	0.009	−0.05(0.67) [−0.34,0.25]	0.001	0.03(0.69) [−0.14,0.20]	0.000	−0.15(0.20) [−0.42,0.12]	0.001	−0.04(0.66) [−0.24,0.16]	0.001	−0.06(0.69) [−0.45,0.32]	0.000		
AU_M	0.27(0.00) [0.13,0.41]	0.014	−0.14(0.21) [−0.38,0.11]	0.001	0.09(0.31) [−0.09,0.26]	0.002	0.02(0.85) [−0.25,0.30]	0.000	0.05(0.63) [−0.15,0.25]	0.002	0.16(0.34) [−0.72, 1.03]	0.002		
Per_M	0.09(0.19) [−0.05,0.23]	0.001	−0.05(0.64) [−0.30,0.19]	0.000	0.01(0.95) [−0.19,0.20]	0.000	−0.13(0.28) [−0.45,0.20]	0.001	−0.16(0.12) [−0.37,0.04]	0.003	0.14(0.41) [−0.45,0.73]	0.001		

	**School satisfaction**				**Self-satisfaction**						**Global satisfaction**			
	**Intercept**	***sr*^2^**	**Slope**	***sr*^2^**	**Time 1**	***sr*^2^**	**Time 2**	***sr*^2^**	**Time 3**	***sr*^2^**	**Intercept**	***sr*^2^**	**Slope**	***sr*^2^**

SP	0.08(0.07) [−0.01,0.18]	0.003	−0.10(0.15) [−0.23,0.04]	0.003	0.26(0.00) [0.17,0.34]	0.047	0.25(0.00) [0.17,0.33]	0.043	0.18(0.00) [0.09,0.26]	0.024	0.08(0.10) [−0.02,0.18]	0.002	−0.09(0.16) [−0.23,0.04]	0.003
DEP	0.27(0.00) [0.17,0.37]	0.035	−0.12(0.08) [−0.27,0.02]	0.003	0.08(0.08) [−0.01,0.16]	0.004	0.03(0.51) [−0.06,0.12]	0.001	0.09(0.04) [0.002,0.19]	0.006	0.14(0.01) [0.03,0.24]	0.013	0.06(0.38) [−0.09,0.21]	0.002
ES	0.14(0.02) [0.02,0.27]	0.007	−0.01(0.95) [−0.19,0.18]	0.000	0.15(0.01) [0.04,0.26]	0.009	0.28(0.00) [0.17,0.39]	0.032	0.22(0.00) [0.09,0.35]	0.020	0.01(0.87) [−0.13,0.15]	0.001	0.27(0.00) [0.09,0.45]	0.015
IR	0.16(0.01) [0.04,0.29]	0.009	−0.05(0.54) [−0.24,0.13]	0.000	−0.08(0.12) [−0.20,0.03]	0.003	−0.12(0.02) [−0.25, −0.001]	0.007	−0.10(0.09) [−0.22,0.02]	0.006	0.29(0.00) [0.13,0.44]	0.022	−0.24(0.01) [−0.43, −0.06]	0.011
AN_F	0.02(0.80) [−0.15,0.19]	0.000	0.06(0.58) [−0.17,0.29]	0.000	0.10(0.13) [−0.05,0.24]	0.003	0.16(0.02) [0.04,0.28]	0.008	0.06(0.45) [−0.09,0.21]	0.000	0.01(0.90) [−0.15,0.17]	0.000	−0.14(0.22) [−0.35,0.08]	0.002
AU_F	0.16(0.048) [0.004,0.31]	0.006	0.07(0.57) [−0.15,0.29]	0.001	20(0.01) [0.03,0.37]	0.009	0.12(0.11) [−0.02,0.26]	0.003	0.14(0.08) [−0.02,0.30]	0.004	0.17(0.05) [−0.02,0.36]	0.004	−0.12(0.31) [−0.38,0.14]	0.001
Per_F	−0.01(0.91) [−0.18,0.16]	0.000	−0.11(0.37) [−0.38,0.29]	0.002	0.03(0.71) [−0.14,0.20]	0.000	0.04(0.66) [−0.12,0.19]	0.000	−0.03(0.72) [−0.20,0.14]	0.001	0.05(0.60) [−0.15,0.24]	0.000	−0.16(0.20) [−0.44,0.12]	0.002
AN_M	−0.12(0.09) [−0.29,0.05]	0.004	0.05(0.65) [−0.17,0.28]	0.001	−0.01(0.84) [−0.16,0.13]	0.000	−0.11(0.10) [−0.24,0.02]	0.004	−0.08(0.32) [−0.22,0.07]	0.001	0.04(0.58) [−0.12,0.21]	0.000	0.01(0.90) [−0.20,0.23]	0.000
AU_M	0.14(0.06) [0.00,0.29]	0.004	−0.08(0.48) [−0.28,0.12]	0.001	0.09(0.19) [−0.06,0.25]	0.002	0.11(0.14) [−0.04,0.25]	0.003	0.06(0.41) [−0.08,0.21]	0.001	0.14(0.08) [−0.04,0.32]	0.004	−0.01(0.92) [−0.24,0.22]	0.000
Per_M	−0.14(0.07) [−0.30,0.01]	0.003	0.13(0.29) [−0.12,0.37]	0.002	−0.01(0.89) [−0.18,0.15]	0.000	−0.08(0.30) [−0.23,0.07]	0.002	−0.07(0.42) [−0.23,0.10]	0.001	0.12(0.18) [−0.07,0.30]	0.002	−0.08(0.52) [−0.32,0.17]	0.001

*SP, Social Potency; DEP, Dependability; ES, Emotional Stability; IR, Interpersonal Relatedness; An_F, Paternal Authoritarian Style; Au_F, Paternal Authoritative Style; Per_F, Paternal Permissive Style; An_M, Maternal Authoritarian Style; Au_M, Maternal Authoritative Style; Per_M, Maternal Permissive Style; *sr*^2^, squared semi-partial correlations. *p*-Values were shown within the parentheses. The bootstrapping 5000 results of 95% confidence intervals were shown within the brackets.*

Results indicated that dependability, interpersonal relatedness, and paternal and maternal authoritative styles positively predicted the initial level of family satisfaction, whereas paternal and maternal authoritarian style negatively predicted the initial level of family satisfaction. As for friend satisfaction, social potency, interpersonal relatedness, and paternal authoritative style positively predicted adolescents’ initial level of friend satisfaction, whereas paternal authoritarian style negatively predicted the initial level of friend satisfaction. The slope of friend satisfaction was positively predicted by emotional stability and paternal authoritarian style, whereas negatively predicted by social potency. The initial level of health satisfaction was positively predicted by social potency and emotional stability. The initial level of school satisfaction was positively predicted by dependability, emotional stability, interpersonal relatedness, and paternal authoritative style. The initial level of global satisfaction was positively predicted by dependability and interpersonal relatedness. The slope of global satisfaction was positively predicted by emotional stability, whereas negatively predicted by interpersonal relatedness.

**FIGURE 1:**
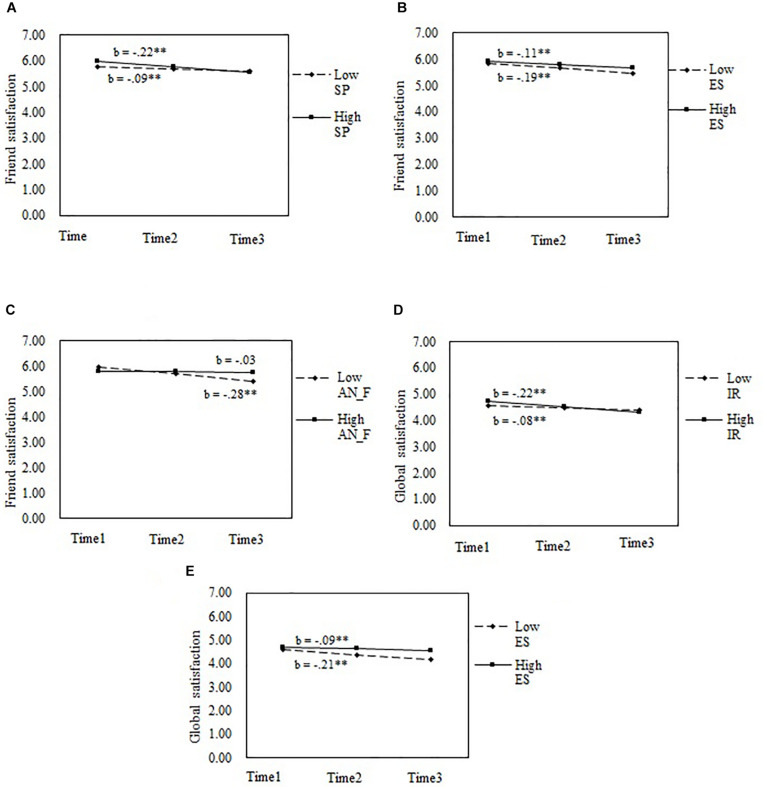
Moderating effects of personality and parenting styles on the growth of life satisfaction from Grade 6 to Grade 8. SP, social potency; ES, emotional stability; IR, interpersonal relatedness; AN_F, paternal authoritarian style. **(A–C)** The moderating effects of social potency, emotional stability, and paternal authoritarian style on growth of friend satisfaction, respectively. **(D,E)** The moderating effects of interpersonal relatedness and emotional stability on growth of global satisfaction, respectively. ^∗^*p* < 0.05; ^∗∗^*p* < 0.01.

These results indicated that social potency, emotional stability, and paternal authoritarian style moderated the growth of friend satisfaction; emotional stability and interpersonal relatedness moderated the growth of global satisfaction. I then conducted simple slope analyses to examine the moderating effects. As presented in Figure 1, students with higher social potency (+ 1 *SD*), lower emotional stability (−1 *SD*), or perceived lower paternal authoritarian style (−1 *SD*) displayed sharper decreasing trends in friend satisfaction than did those with lower social potency (−1 *SD*), higher emotional stability (+ 1 *SD*), or perceived higher paternal authoritarian style (+ 1 *SD*) (see Figures 1A–C, respectively). Students with higher interpersonal relatedness (+ 1 *SD*) or lower emotional stability (−1 *SD*) displayed sharper decreasing trends in global satisfaction than did those with lower interpersonal relatedness (−1 *SD*) or higher emotional stability (+ 1 *SD*) (see Figures 1D, E, respectively).

The regression results of self-satisfaction at three time points on parenting styles and personality were reported in Table 4. Results showed that social potency, emotional stability and paternal authoritative style positively predicted self-satisfaction at Time 1 and totally complained 25% variance. Self-satisfaction at Time 2 (*R*^2^ = 0.24) was positively predicted by paternal authoritarian style, social potency, and emotional stability, whereas negatively predicted by interpersonal relatedness. Self-satisfaction at Time 3 (*R*^2^ = 0.19) was positively predicted by social potency, dependability, and emotional stability.

## Discussion

The present longitudinal study explored the developmental trajectories of life satisfaction and investigated the influences of both maternal and paternal parenting styles and both universal independent personality and Chinese indigenous interdependent personality on life satisfaction in a Chinese adolescent sample.

### The Development of Life Satisfaction in Chinese Adolescents

Consistent with our hypothesis, adolescents displayed decreasing trends in both global and domain-specific life satisfactions as the school year progressed. This result reinforced previously reported evidence from Hong Kong (e.g., Shek and Liang, 2018), Taiwan (e.g., Jhang, 2018), mainland China (e.g., Wang and Zhang, 2012), and other countries (e.g., Marquez and Long, 2020). Such decreasing trends may be due to the high expectations (Shoshani and Slone, 2013) and schoolwork pressure (Cosma et al., 2020) during the middle school years in this highly formative era. The inconsistent findings with the stable pattern shown in mainland Chinese high school students (Nie et al., 2019) may indicate that the developmental trajectories in life satisfaction during early adolescence and late adolescence are different (e.g., Chen, 2020).

### Life Satisfaction and Personality

The results indicated that four CPAI dimensions significantly predicted both global and domain-specific life satisfaction in Chinese adolescents. The functions of these personality factors varied across different domains of life satisfaction. Such results supported the view that the global and domain-specific life satisfaction are distinguishable and may have different relationships with personality (Weber and Huebner, 2015).

Specifically, consistent with previous studies (e.g., Xie et al., 2016), adolescents who scored higher on social potency reported higher levels of satisfaction in friend and health domains at the initial stage and higher self-satisfaction at all three time points; no significant influence of social potency on the global life satisfaction was viewed (e.g., Chen et al., 2006). Unexpectedly, I found that individuals with higher levels of social potency experienced shaper decrease in friend satisfaction over time. Nevertheless, this result is similar to Li et al.’s (2019) research findings that Chinese adolescents with higher levels of social potency experienced increased loneliness from Grade 6 to Grade 8. It is possible that individuals who are extravertive and like to take the lead in making decisions would maintain larger social networks and thus have higher levels of friend satisfaction at the initial stage (e.g., Moran and Weiss, 2006; Harris and Vazire, 2016). However, as adolescents grow up, “good friends” are not only those who spend time together, but more those who can chat about beliefs, values, and ideologies with each other (Brown, 2004). Thus, it is conceivable that sharper decreasing trends in friend satisfaction for those with higher levels of social potency would be viewed during the research period.

Dependability was found to positively predict the initial levels of family, school, and global life satisfaction, which is in agreement with previous research (e.g., Chen et al., 2006; Xie et al., 2016). For example, Xie et al. (2016) reported positive associations between dependability and the global life satisfaction as well as the satisfaction in family and school domains. Dependable people who are high in responsibility and have clear life meaning tend to plan ahead and pursue meaningful goals (Cheung et al., 2008), and thus would be more likely to be relied on and have higher life satisfaction (Chen et al., 2006; Ho et al., 2010). Our current finding that dependability only predicted self-satisfaction at Time 3 may indicate that traits like responsibility and meaning in life are more important in determining one’s self-satisfaction at higher grades comparing to their contributions at lower grades.

Given the overlapping between dependability and conscientiousness (Cheung et al., 2001), the findings of present study are also, to some extent, consistent with previous studies in which a positive association between conscientiousness and life satisfaction was reported (e.g., Suldo et al., 2015; Weber and Huebner, 2015). For example, Weber and Huebner (2015) found that conscientiousness was positively related to satisfaction in family life and school experiences domains in US adolescents. With a sample of US high school students, Suldo et al. (2015) reported that conscientiousness positively predicted global life satisfaction.

Higher levels of emotional stability were associated with higher initial levels of satisfaction in health and school domains as well as higher self-satisfaction at all three time points, which substantiates previous empirical studies (e.g., Xie et al., 2016; Tian et al., 2017; Cohrdes and Mauz, 2020). For example, Tian et al. (2017) reported a positive association between emotional stability and subjective well-being in school (i.e., school satisfaction and school affect) in Chinese adolescent students. Although adolescents generally displayed a decrease trend in life satisfaction in the current study, it was found that highly emotionally stable individuals declined less from Grade 6 to Grade 8. These results suggested that emotional stability could serve as a protective factor that helps adolescents cope with stressful situations (e.g., Ho et al., 2013).

The positive predictions from interpersonal relatedness on the initial levels of adolescents’ family, friend, and school satisfactions are consistent with previous findings in Chinese adolescents. For example, Xie et al. (2016) found significant and positive influence of interpersonal relatedness on family, friend, and school satisfaction in a sample of junior secondary school students. These results provided empirical support for the idea that the interpersonal relationship orientation is associated with the quality of social relationships (Cheung et al., 2001) and leads to the satisfaction in interaction with others (Xie et al., 2016). Higher levels of interpersonal relatedness were also related to higher initial levels in global life satisfaction. This result is in agreement with previous research in which a positive association between interpersonal relatedness and adolescents’ global life satisfaction was reported (e.g., Ho et al., 2008; Xie et al., 2016). Such findings supported the idea that the emphasis on relationships with others can help to foster individuals’ life satisfaction (Blanca et al., 2018).

However, contrary to our hypothesis, individuals with higher levels of interpersonal relatedness experienced a shaper decrease in global life satisfaction over time and lower levels of self-satisfaction at Time 2. This result suggested that the tendency to maintain useful ties, avoid conflict, and contribute to the collective over the individual goals (features of interpersonal relatedness) may sometimes have negative consequences because individuals may give up their own interests and maintain even harmful relationships (Kim et al., 2018). The contribution of interpersonal relatedness to Chinese adolescents’ life satisfaction highlighted the importance of considering the indigenous personality when exploring factors that influence adolescents’ development in the specific cultural context.

### Life Satisfaction and Parenting Styles

Regarding the variations in life satisfaction as a function of maternal and paternal parenting styles, the present results overall indicated that parenting styles were related to domain-specific life satisfaction. However, in contrast with literature showing positive link between global life satisfaction and parental authoritative style in Western and non-Western cultures, and negative link between global life satisfaction and parental authoritarian style in Western cultures and positive link between global life satisfaction and parental authoritarian style in Chinese Context (e.g., Milevsky et al., 2007; Xie et al., 2016; Gherasim et al., 2017), parenting styles in the present study did not influence the global life satisfaction when the effect of adolescents’ personality is considered. One possible reason is that most previous studies did not consider the influence of individuals’ personality when exploring the influence of parenting styles on life satisfaction (e.g., Abubakar et al., 2015). Results in the current study implied that the influence of parenting on student development becomes less important as individuals’ demand for independence and autonomy increase during adolescence (Inguglia et al., 2015), and that personality traits were better predictors of life satisfaction than situational factors (Diener et al., 1999).

In the present study, adolescents who perceived their mothers or fathers as more authoritative reported higher family satisfaction at the initial stage, and those who perceived their mothers or fathers as more authoritarian reported lower family satisfaction at the initial stage. These findings are consistent with the previous studies that were conducted in Western contexts, showing that authoritative parenting style is associated with happier family life and authoritarian is related to lower family satisfaction (e.g., Givertz and Segrin, 2014). Such similar results may indicate that whether in Western or in non-Western contexts, authoritative parenting is preferred than authoritarianism in achieving a child perceived happy family life. An alternative explanation is that the present sample was recruited from Shanghai that has a relatively high exposure to the Western cultural context, and thus would display a similar relationship pattern with the findings in Western context. It was also noted that on family satisfaction, paternal and maternal parenting styles have a similar function.

In addition, paternal authoritative style was found to be associated with higher levels of friend, school, and self-satisfaction at the initial stage. These results were in accordance with the findings of previous studies in which authoritative style was shown to play an important role in adolescents’ interpersonal management (e.g., Shalini and Acharya, 2013), avoidance of social withdrawal (e.g., Sandhu and Sharma, 2015), school adjustment (e.g., Pinquart and Kauser, 2018), and general self-efficacy (e.g., Tam et al., 2012).

It should be noted that adolescents who perceived lower paternal authoritarian style reported higher levels of friend satisfaction at the initial stage and sharper decrease trends over 2 years. Although this result was unexpected, similar observations were made by Di Maggio and Zappulla (2014). In their study, Italian high school students (with the similar age to the participants in the present study at Time 3) who perceived their fathers as authoritarian reported higher levels of satisfaction with friends than those with authoritative fathers.

It was also found that adolescents who perceived higher paternal authoritarian parenting would be more likely to be satisfied of themselves. These findings hinted that lower authoritarian parenting may not always be a good thing for Chinese adolescents’ life satisfaction (Chao, 1994). Specifically, unlike in Western cultures where authoritarianism is construed as rejection, in the Chinese context that Confucian philosophy is highlighted, the authoritarian parenting may be perceived as training and monitoring. Chinese parents who engage the authoritarian parenting would supervise, monitor, and guide their children’s behaviors to fit the rules and standards of the society. Such guidance and monitoring are beneficial for the formation of children’s adaptive working model of self, such as seeing oneself as lovable, competent and worthy (Li et al., 2016). This result is also consistent with previous research which showing that parental behavioral control associated with higher life satisfaction among Chinese adolescents (e.g., Zhu and Shek, 2021).

## Theoretical and Practical Implications

The current three-wave longitudinal study provided at least three aspects of theoretical contributions. First, the findings provided more empirical evidence of the development trajectories in life satisfaction among early adolescents, which has been rarely examined in the context of mainland China. Second, this study highlighted the impact of culture-specific personality (i.e., interpersonal relatedness) on the development of adolescents’ life satisfaction above and beyond the universal independent personality factors (i.e., social potency, emotional stability, and dependability). Such results suggested that including culture-specific personality in research might provide a better prediction of developmental outcomes than only considering universal personality factors. Third, the findings provide further evidence for the long-term influence of parenting styles and add value to the research field by revealing how paternal and maternal styles may affect the trajectories in each specific aspects of life satisfaction among early adolescents, respectively. The differences in functions of paternal and maternal parenting styles highlighted the importance of examining the consequences of parenting practices separately for mothers and fathers.

The current study also has possible implications for potential prevention and intervention programs aiming at promoting early adolescent life satisfaction. First, personality assessment, particularly relevant with indigenous personality attributes, may help with the identification of adolescents who are at risk of life dissatisfaction. The close relationship between personality and life satisfaction suggests that early intervention efforts are warranted for adolescents at Grade 6 with lower levels of social potency, dependability, emotional stability, and interpersonal relatedness to avoid maladaptive functioning which was progressed from low life satisfaction (e.g., Proctor et al., 2009). Furthermore, given that adolescents with higher levels of social potency and interpersonal relatedness appeared to decline sharper in life satisfaction, although high life satisfaction at Grade 6 were displayed, attention should also be paid to those who seemed untargeted at Grade 6.

Second, interventions are suggested to be designed to help with adolescents’ personality development. Although personality is relatively stable throughout the life span, there is evidence that personality is changeable and malleable due to significant life events or changing environments (e.g., Specht et al., 2011). Programs focusing on personality would be helpful to enhance the life satisfaction of adolescents. Because the influence of personality on life satisfaction was different across varied life domains, intervention could be designed according to which specific domain of satisfaction is targeted. For example, in order to promote satisfaction in social life (i.e., family, friend, and school satisfaction) and help those with social adjustment problems, professionals and teachers could encourage and cultivate adolescents to be more interpersonal-relationship orientated.

Third, given that authoritative parenting is linked to higher levels of life satisfaction, family interventions and family education program are suggested to employ to improve the practice of authoritative parenting. Moreover, most work on parenting styles has combined maternal and paternal styles (e.g., Xiang et al., 2017), or only focused on the effects of maternal parenting styles (e.g., Gherasim et al., 2017), whereas the present study showed that, compared with maternal parenting, paternal parenting seemed to play a more important role in Chinese adolescents’ lives, especially in the friend, school, and self domains of life satisfaction. Therefore, intervention programs aiming at promoting adolescent life satisfaction should not only incorporate components related to maternal parenting behaviors, but also pay attention to fathers’ involvement and encourage and cultivate fathers to adopt the authoritative parenting style.

## Limitations and Future Directions

The present study has several limitations that might suggest directions for future research. First, although the study was a three-wave longitudinal design that lasted 2 years, it only explored the development of life satisfaction in early adolescents. To capture the characteristics during the whole adolescence stage, future longitudinal studies should continue exploring the change patterns of adolescents’ life satisfaction over a longer time frame. Second, only adolescents’ self-report measures were employed in the present study. Although I have checked the common method bias, the self-reported data may still have some limitations, such as the social desirability bias. Parent-report, teacher-report, and peer-report measures could be employed to supplement students’ reports in future research. Third, the sample in the present study was from one city in mainland China, which may be distinctive to other cities due to different economic and educational backgrounds. Future research could examine more regions with different Chinese local cultures. Finally, the self-satisfaction dimension in this study only achieved the partial scalar invariance, which indicates that mean differences of self-satisfaction did not capture all mean differences in shared variance of the items measuring this dimension. To reduce chances of running into such problems, future studies should revise the self-satisfaction items and improve its psychometric properties.

## Data Availability Statement

The raw data supporting the conclusions of this article will be made available by the authors, without undue reservation.

## Ethics Statement

The studies involving human participants were reviewed and approved by Shanghai Normal University. Written informed consent to participate in this study was provided by the participants’ legal guardian/next of kin.

## Author Contributions

ML substantially contributed to the conception and the design of the work, acquisition of data and analyzed and interpreted the data, and prepared the draft and finalize it. The author approved the final version of the manuscript for submission.

## Conflict of Interest

The author declares that the research was conducted in the absence of any commercial or financial relationships that could be construed as a potential conflict of interest.

## Publisher’s Note

All claims expressed in this article are solely those of the authors and do not necessarily represent those of their affiliated organizations, or those of the publisher, the editors and the reviewers. Any product that may be evaluated in this article, or claim that may be made by its manufacturer, is not guaranteed or endorsed by the publisher.

## Appendix

**APPENDIX 1:** Measurement Invariance Tests of Life Satisfaction Scale.

	**Model**	**χ^2^**	***df***	**RMSEA**	**SRMR**	**CFI**	**Δχ^2^(Δ*df*)**	**ΔRMSEA**	**ΔCFI**
Family satisfaction	configural invariance	17.32	18	0.000	0.022	1.000			
	metric invariance	18.35	22	0.000	0.024	1.000	1.03(4)	0.000	0.000
	scalar invariance	21.63	26	0.000	0.027	1.000	4.31(8)	0.000	0.000
Friend satisfaction	configural invariance	131.61	51	0.047	0.028	0.970			
	metric invariance	132.48	57	0.043	0.030	0.972	0.87(6)	–0.004	0.002
	scalar invariance	136.20	63	0.040	0.031	0.973	4.59(12)	–0.007	0.003
Health satisfaction	configural invariance	270.61	51	0.078	0.044	0.913			
	metric invariance	277.82	57	0.074	0.048	0.913	7.21(6)	–0.004	0.000
	scalar invariance	282.17	63	0.070	0.048	0.914	11.56(12)	–0.008	0.001
School satisfaction	configural invariance	95.83	23	0.067	0.029	0.971			
	metric invariance	106.39	27	0.064	0.047	0.968	1.59^∗^(4)	–0.003	–0.003
	scalar invariance	108.47	31	0.059	0.048	0.969	12.64(8)	–0.008	–0.002
Self-satisfaction	configural invariance	20.41	15	0.023	0.024	0.998			
	metric invariance	27.84	19	0.026	0.033	0.997	7.43(4)	0.003	–0.001
	scalar invariance	92.08	23	0.065	0.053	0.978	71.67^∗∗^(8)	0.042^b^	−0.020^b^
	partial scalar invariance^a^	33.47	21	0.029	0.034	0.996	13.06^∗^(6)	0.003	–0.001
Global satisfaction	configural invariance	371.61	72	0.077	0.054	0.907			
	metric invariance	381.53	80	0.073	0.058	0.907	9.92(8)	–0.004	0.000
	scalar invariance	404.09	88	0.071	0.060	0.902	32.48^∗∗^(16)	–0.006	–0.005

*CFI, Comparative Fit Index; RMSEA, Root Mean Square Error of Approximation; SRMR, Standardized Root Mean Square Residual. To determine significant differences between these two models at least two of the following three criteria had to be matched: Δχ^2^ significant at *p* < 0.05, ΔCFI ≥ 0.01, and ΔRMSEA ≥ 0.015 (Negru-Subtirica et al., 2015).*

***p* < 0.05; ***p* < 0.01.*

*^*a*^partial scalar invariance with two intercepts freed.*

*^*b*^ΔCFI or ΔRMSEA higher than cutoffs.*
